# Unlocking molecular mechanisms and identifying druggable targets in matched-paired brain metastasis of breast and lung cancers

**DOI:** 10.3389/fimmu.2023.1305644

**Published:** 2023-12-12

**Authors:** Shiva Najjary, Willem de Koning, Johan M. Kros, Dana A. M. Mustafa

**Affiliations:** Department of Pathology and Clinical Bioinformatics, The Tumor Immuno-Pathology Laboratory, Erasmus University Medical Center, Rotterdam, Netherlands

**Keywords:** molecular mechanisms, brain metastasis, breast cancer, lung adenocarcinoma, gene expression

## Abstract

**Introduction:**

The incidence of brain metastases in cancer patients is increasing, with lung and breast cancer being the most common sources. Despite advancements in targeted therapies, the prognosis remains poor, highlighting the importance to investigate the underlying mechanisms in brain metastases. The aim of this study was to investigate the differences in the molecular mechanisms involved in brain metastasis of breast and lung cancers. In addition, we aimed to identify cancer lineage-specific druggable targets in the brain metastasis.

**Methods:**

To that aim, a cohort of 44 FFPE tissue samples, including 22 breast cancer and 22 lung adenocarcinoma (LUAD) and their matched-paired brain metastases were collected. Targeted gene expression profiles of primary tumors were compared to their matched-paired brain metastases samples using nCounter PanCancer IO 360™ Panel of NanoString technologies. Pathway analysis was performed using gene set analysis (GSA) and gene set enrichment analysis (GSEA). The validation was performed by using Immunohistochemistry (IHC) to confirm the expression of immune checkpoint inhibitors.

**Results:**

Our results revealed the significant upregulation of cancer-related genes in primary tumors compared to their matched-paired brain metastases (adj. *p* ≤ 0.05). We found that upregulated differentially expressed genes in breast cancer brain metastasis (BM-BC) and brain metastasis from lung adenocarcinoma (BM-LUAD) were associated with the metabolic stress pathway, particularly related to the glycolysis. Additionally, we found that the upregulated genes in BM-BC and BM-LUAD played roles in immune response regulation, tumor growth, and proliferation. Importantly, we identified high expression of the immune checkpoint VTCN1 in BM-BC, and VISTA, IDO1, NT5E, and HDAC3 in BM-LUAD. Validation using immunohistochemistry further supported these findings.

**Conclusion:**

In conclusion, the findings highlight the significance of using matched-paired samples to identify cancer lineage-specific therapies that may improve brain metastasis patients outcomes.

## Introduction

The incidence of brain metastasis (BM) is rising. Improved therapeutic methods to control the primary tumors have led to longer survivals, but have also increased the incidence of late metastases to brain (1, 2). Despite the development of novel targeted therapies, BM remains a devastating complication of cancer. BM patients exhibit poor prognosis due to no effective long-term therapy (3, 4). BM from lung and breast cancer exhibits distinct patterns in the disease course. In lung cancer, BM typically develops within a few months after diagnosis. On the other hand, in breast cancer, metastasis often occurs in one organ before it spreads to other organs, with brain metastasis typically being a late event in the course of disease (5). The median overall survival of the patients diagnosed with BM is approximately 4-15 months in lung cancer and 6-18 months in breast cancer following treatment (2, 6). Due to the limited effectiveness of current therapies and the poor prognosis associated with BM (7–9), it is crucial to identify related genes and molecular mechanisms involved. Understanding these mechanisms can aid in the identification of new therapeutic targets and potentially improve patient survival. The signaling pathways and gene expression profiles in metastatic cancer cells play a significant role in in the formation of organ-specific metastasis. Specific gene expression patterns may dictate tendency for specific targeting specific sites, such as the brain. The unique physiological barriers in the brain, including the blood-brain-barrier (BBB), is hypothesized to limit the ability of metastatic tumor cells to infiltrate the brain (10). Site-specific metastasis may also reflect tissue-specific migration and proliferation signatures that contribute to the differential ability of tumor cells to metastasize and proliferate in specific organs (11, 12). Compared to other sites of cancer metastasis, the normal brain tissue has relatively low collagen content, lower oxygen tension, and high glucose metabolism, which may affect the survival and proliferation of disseminated tumor cells in the brain (13, 14). So far, the molecular signatures and expression patterns involved in brain metastasis remain largely unknown. Employing matched-paired samples from both primary tumors and brain metastases constitutes a distinctive and precise research approach, enabling a thorough investigation of the association between gene expression signatures and metastatic development. Despite its potential, few studies have utilized this method, underscoring the uniqueness of the presented research. Primary lung cancer and their matched-paired were used in previous studies to generate gene expression signature (15). Similarly, 16 primary breast cancers and their matched-paired brain metastases were used to generate gene expression profiles in order to assess clinic-pathological markers, immune-related gene signatures, and identifying therapeutic targets for brain metastases (16). In addition, 21 breast cancer and their matched-paired samples were used previously to perform TrueSeq RNA-sequencing and determine clinically actionable BM target genes (17).

The aim of the present study was to investigate the mechanisms operative in the primary tumors and their matched-paired brain metastasis. The utilization of matched-paired samples provides a unique opportunity to investigate shared molecular characteristics and distinctions, advancing our understanding and potential treatments for malignancies. We compared gene expression profiles of primary tumors with their matched-paired BM. Subsequently, we thoroughly examined the upregulated genes in BM of both tumor types to identify shared and distinctive predominant molecular features in BM. We validated the identified druggable targets by using IHC in the matched-paired samples. The collective findings from our research have the potential to enhance the treatment strategies for patients with BM.

## Materials and methods

### Tissue samples selection and clinicopathological data

**Figure 1:**
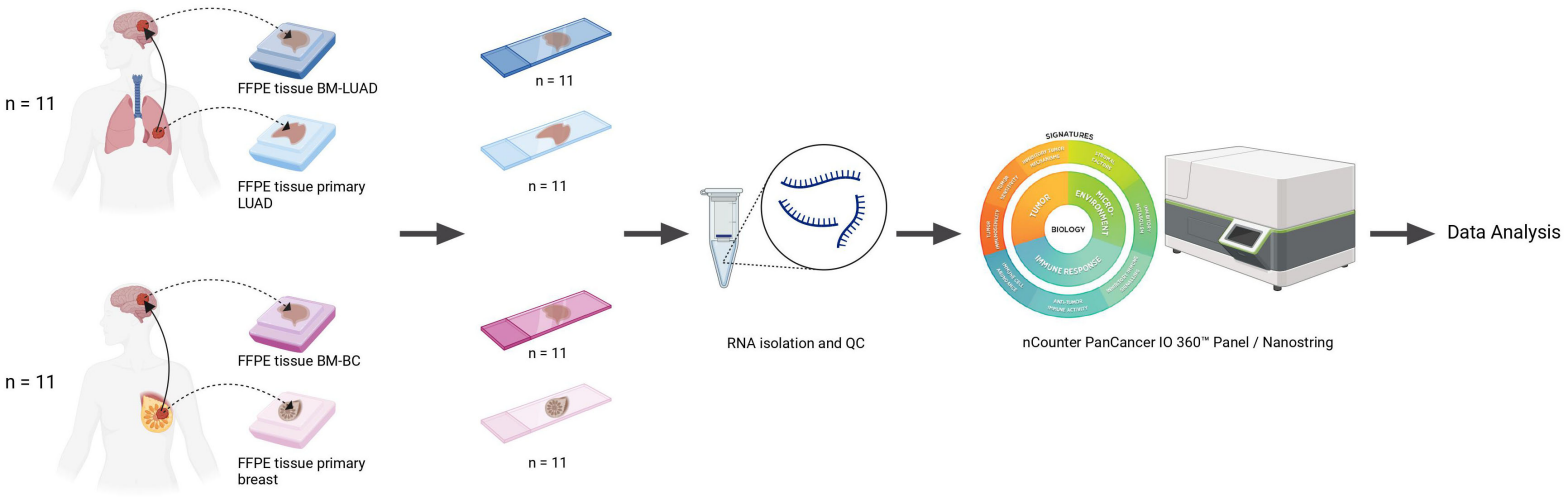
Schematic overview of the study.

A set of 44 Formalin-fixed, paraffin-embedded (FFPE) tissue samples of primary tumors and their matched-paired brain metastases was used, including 11 pairs of lung adenocarcinoma and 11 pairs of breast cancer (**Figure 1**). The clinicopathological characteristics of patients are summarized in **Table 1**. None of the patients in either group received therapy or treatment with corticosteroids within the 6–12 months prior to brain metastasis surgery. Treatment options following surgical removal of the brain metastasis included radiotherapy, chemotherapy, stereotactic radiotherapy (SRT), and whole brain radiotherapy (WBRT). More than half of the patients with LUAD were males and the majority of patients had a smoking history. The median age at diagnosis of the brain metastases was 48 years for patients with breast cancer and 64 years for patients with LUAD. This study was approved by the Medical Ethics Committee of the Erasmus Medical Center, Rotterdam, the Netherlands, and was carried out in adherence to the Code of Conduct of the Federation of Medical Scientific Societies in the Netherlands (MEC 02·953 & MEC-2020–0732).

**Table 1:** Clinical characteristics of patients.

Characteristics	No.	%
Total samples	44	100
Cancer type		
Primary breast cancer	11	25
BM-BC	11	25
Primary lung adenocarcinoma	11	25
BM-LUAD	11	25
Lung adenocarcinoma		
Median age at diagnosis with primary tumor, years (range)	57 (37-69)	
Median age at diagnosis with brain metastasis tumor, years (range)	64 (46-74)	
Sex		
Male	6	54.5
Female	5	45.5
Histology of primary tumor and their matched-paired brain metastases		
Adenocarcinoma	22	100
Smoking status		
Never smoking	1	9.1
Former smoker	5	45.5
Smoking	4	36.4
Unknown	1	9.1
Treatment for primary tumor		
Surgery	3	27.3
Surgery & Chemotherapy	1	9.1
surgery & Radiotherapy	2	18.2
Surgery & Chemotherapy & Radiotherapy	2	18.2
surgery & Radiochemotherapy	3	27.3
Treatment after surgery of BM		
No treatment	1	9.1
Radiotherapy	6	54.4
SRT	1	9.1
WBRT	2	18.2
Other	1	9.1
Breast cancer		
Median age at diagnosis with primary tumor, years (range)	47 (40-74)	
Median age at diagnosis with brain metastasis tumor, years (range)	48 (44-74)	
Sex		
Female	11	100
Histology of primary tumor		
ER/PR+	2	18.2
ER/HER2+	1	9.1
HER2+	1	9.1
TNBC	5	45.5
Unknown	2	18.2
Histology of matched-paired brain metastases		
ER+	3	16.7
PR+	2	11.1
ER/PR+	1	5.6
HER2+	2	11.1
TNBC	2	11.1
Unknown	1	5.6
Treatment for primary tumor		
Surgery	2	18.2
Surgery & Chemotherapy	3	27.3
Surgery & Chemotherapy & Radiotherapy	6	54.5
Treatment after surgery of BM		
Radiotherapy	5	45.5
Chemotherapy	2	18.2
Radiotherapy & Chemotherapy	2	18.2
Other	2	18.2

ER, estrogen receptor; PR, progesterone receptor; HER2, human epidermal growth factor 2; TNBC, triple-negative breast cancer; SRT, Stereotactic radiotherapy; WBRT, Whole brain radiotherapy.

### RNA isolation and quality control

RNA isolation and QC were performed following our previously described protocol (18). In brief, 5 μm H&E tissue sections from each sample were examined by a pathologist. Total RNA was isolated from 10-12 sections of 10 μm using the RNeasy FFPE kit (Qiagen, Hilden, Germany) according to the supplier’s instructions. The extracted RNA was stored in RNase/DNase-free water at -80°C. The quality and quantity of the RNA was determined using the Agilent 2100 Bioanalyzer (Santa Clara, CA, USA). RNA degradation was assessed by calculating the percentage of fragments within the range of 300-4000 nucleotides.

### Targeted gene expression profiling using Nanostring^®^ nCounter assay

To measure targeted gene expression profiles, the nCounter PanCancer IO360™ Panel consisting of 750 cancer-related genes and 20 housekeeping genes, was used (Nanostring Technologies, Seattle, WA, United States) (19). Briefly, 300 ng of RNA, up to a maximum of 7 μL, was hybridized with the panel probes for 17 hours at 65°C using a SimpliAmp Thermal Cycler (Applied Biosystems, Foster City, CA, USA). The removal of unannealed probes was performed through the nCounter FLEX system, following the manufacture’s protocol. Gene counts were determined by scanning 490 fields-of-view (FOV). The obtained expression data were imported into the nSolver software (version 4.0), and subsequent analysis was carried out using the Advanced Analysis module (version 2.0). To normalize the raw expression data, the geNorm algorithm was applied, utilizing the 16 most stable housekeeping genes (**Table S1**). Genes were included for further analysis if their expression was higher than the detection limit, which was calculated as the mean of negative controls plus 2 standard deviations.

### Statistical analysis

A simplified negative binomial model, log-linear models, or a mixture of negative binomial models that are embedded within the advanced analysis module, were used to identify differentially expressed genes (DEGs) between pairs of breast cancer and LUAD and their matched-paired brain metastases. To correct for multiple testing, Benjamin-Hochberg method was used and genes with a BH- *P*-value ≤ 0.05 were considered as DEGs. All statistical analyses were performed using R version 4.0.1. The *P*-values were two-sided, and statistical significance was defined as *P*-values ≤ 0.05. Heatmaps were created using Log2-normalized data of significantly expressed genes (adj. *p* ≤ 0.05). Heatmaps of the DEGs were created using the following criteria: |Log2Fold change| > 1.5, adj. *p* ≤ 0.05. Outliers were eliminated using Tukey’s rule (20). To create and visualize heatmaps, the web-based tool Morpheus by the Broad Institute (RRID: SCR_017386) was used. The scaling was performed for each and ranged from 0 to 1.

### Pathways analysis

To assess the differences at the pathway level, two different methods were employed. The first was gene set analysis (GSA) was carried out using the embedded methodology within nSolver software. Pathway scores were calculated using the average expression of genes associated with each pathway. Significant changes in pathway scores between the two groups were determined using the Wilcoxon test, and a *p*-value < 0.05 was considered significant. The second method was by using Gene Set Enrichment Analysis (GSEA) that was performed using the online bioinformatics tool and database, Metascape.org. The NanoString panel was used as the background. DEGs (adj. *p* ≤ 0.05) in BM-BC and BM-LUAD were separately uploaded to the pathway analysis tool, which identified enriched biological pathways and conducted functional enrichment analysis. The obtained results were thoroughly examined and interpreted.

### Immunohistochemistry

Immunohistochemistry (IHC) using the Alkaline phosphatase was performed to investigate the expression of immune checkpoint inhibitors IDO1, VISTA, NT5E, VTCN1 and drug target HDAC3 in all the available samples. Tissue sections of 4μm from each sample were prepared. Primary antibodies were applied and incubated overnight at 4°C, followed by the application of alkaline phosphatase-conjugated secondary antibodies for detection. The details of the antibodies that were used are summarized in (**Table S2**). The stained slides were evaluated and interpreted by a pathologist.

## Results

### Expression of cancer-related genes is higher in the primary tumors than in the matched-paired BM for both LUAD and BC

**Figure 2:**
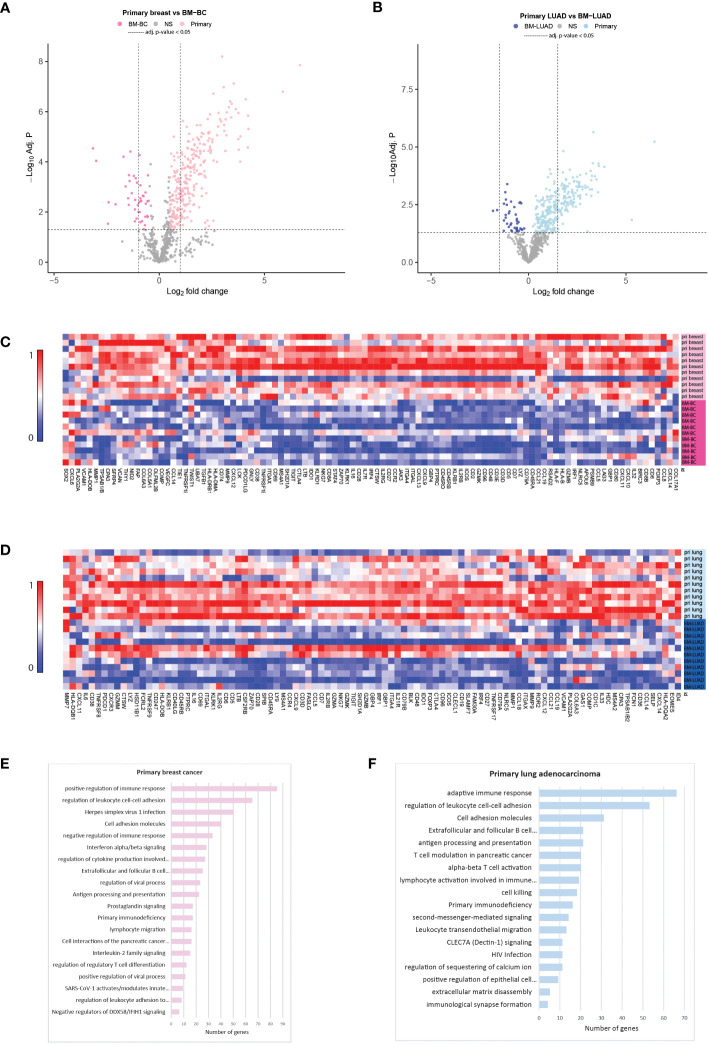
Targeted gene expression analysis between primary tumors with their matched-paired brain metastasis using nCounter PanCancer IO 360™ Panel and gene set enrichment analysis (GSEA) for primary tumors using Metascape.org tool. **(A)** Volcano plot showing differentially expressed genes between primary breast cancer and BM-BC (adj. *p* ≤ 0.05). **(B)** Volcano plot showing differentially expressed genes between primary LUAD and BM-LUAD (adj. *p* ≤ 0.05). **(C)** Heatmap of normalized differentially expressed genes between primary breast and BM-BC (absolute fold change ≥ 1.5; adj. *p* ≤ 0.05). The heatmap is scaled based on each gene. **(D)** Heatmap of normalized differentially expressed genes between primary LUAD and BM-LUAD (absolute fold change ≥ 1.5; adj. *p* ≤ 0.05). The heatmap is scaled based on each gene. **(E)** GSEA of upregulated differentially expressed genes in primary breast cancer (adj. *p* ≤ 0.05). **(F)** GSEA of upregulated differentially expressed genes in primary LUAD (adj. *p* ≤ 0.05).

Comparing the primary breast cancer samples to their matched-paired brain metastases showed that 281 genes were upregulated in primary breast cancers (adj. *p* ≤ 0.05; **Table S3**) and 43 were upregulated in BM-BC (adj. *p* ≤ 0.05; **Table S4**) (**Figures 2A, C**). As to the lung adenocarcinomas 226 genes were upregulated in the primary tumors (adj. *p* ≤ 0.05; **Table S5**) while 20 genes were upregulated in BM-LUAD (adj. *p* ≤ 0.05; **Table S6**) (**Figures 2B, D**). Pathway analysis revealed that the upregulated genes in the primary tumors were associated mainly with cell growth and proliferation (**Figures 2E, F**).

### Different tumor types upregulate distinct genes in their brain metastasis

**Figure 3:**
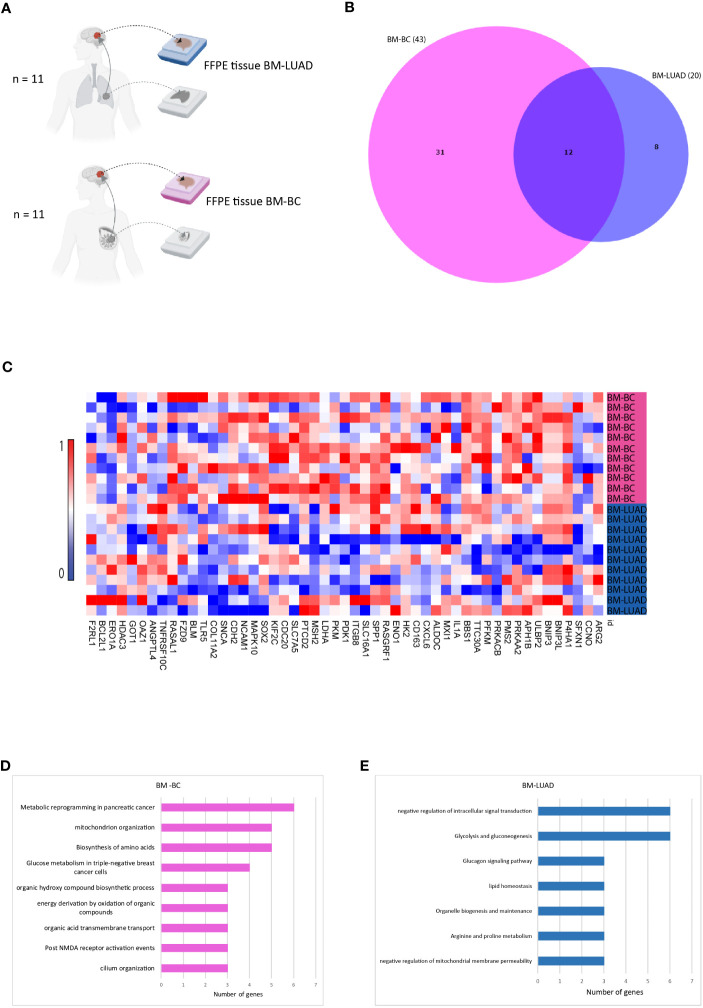
Comparison of upregulated genes in BM-BC and BM-LIUAD. **(A)** Schematic overview of the samples included in the comparison of differentially expressed genes between BM-BC and BM-LIUAD. **(B)** Venn diagram indicating the overlap of differentially expressed genes between BM-BC and BM-LIUAD. **(C)** Heatmap of normalized differentially expressed genes between BM-BC and BM-LUAD (adj. *p* ≤ 0.05). The heatmap is scaled based on each gene. **(D)** GSEA of upregulated differentially expressed genes in BM-BC (adj. *p* ≤ 0.05). **(E)** GSEA of upregulated differentially expressed genes in BM-LUAD (adj. *p* ≤ 0.05).

In order to investigate whether different types of tumors upregulated the same genes after developing brain metastasis, the upregulated genes in BM of both tumor types were compared (**Figure 3A**). The DEGs in BM-BC (n=43) and BM-LUAD (n=20) revealed 12 shared upregulated genes (adj. *p* ≤ 0.05) (**Figure 3B**). Of the other genes 31 were specifically upregulated in BM-BC (adj. *p* ≤ 0.05) and 8 genes in BM-LUAD (adj. *p* ≤ 0.05) (**Figures 3D, C**). Pathway analysis revealed that 6 genes of the commonly upregulated DEGs in BM-BC and BM-LUAD were associated with the metabolic stress pathway (**Figures 3D, E**).

### Common upregulation of the metabolic stress pathway in brain metastases of BC and LUAD

**Figure 4:**
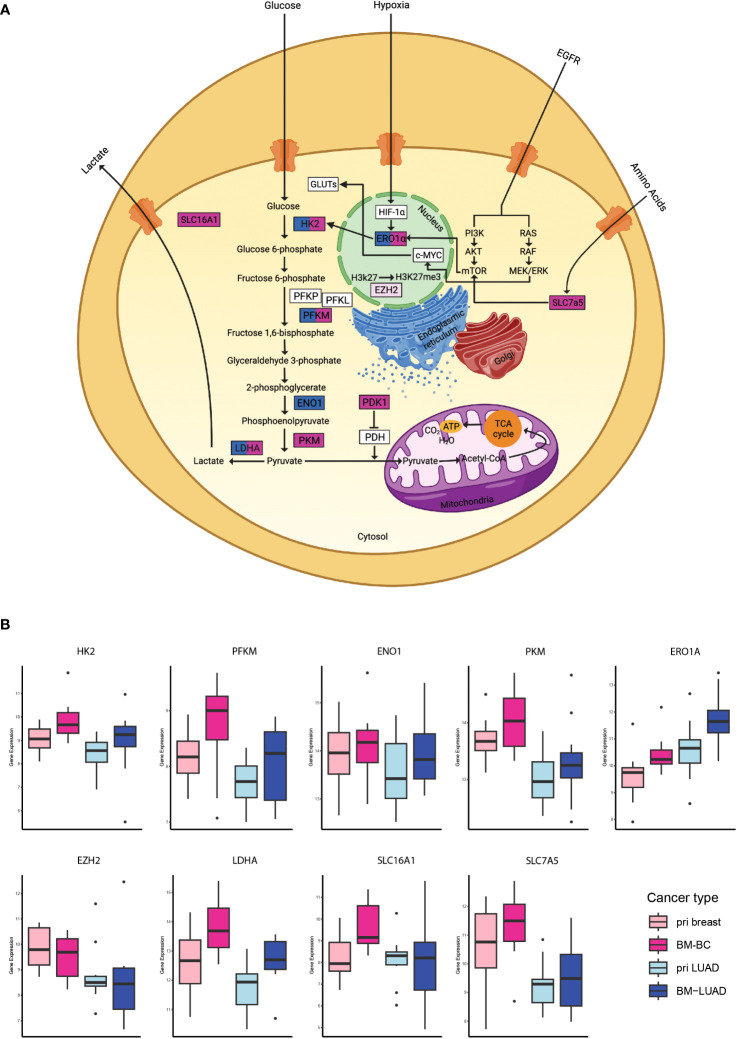
Analysis of the metabolic stress pathway in BM-BC and BM-LIUAD. **(A)** Schematic image of a cell with the differentially expressed genes involved in the metabolic stress and glycolysis. **(B)** Box plots of the differentially expressed genes associated with glycolysis within the metabolic stress pathway in brain metastasis (adj. *p* ≤ 0.05). The word “pri” in the figure refers to primary tumor.

To further investigate the involvement of the upregulated DEGs in brain metastasis, 43 DEGs in BM-BC and 20 DEGs in BM-LUAD were uploaded separately to the GSA and GSEA tools. The results showed that the metabolic stress pathway was enriched in BM with 13 genes in BM-BC and 7 in BM-LUAD. A total of 9 genes of the metabolic stress pathway were found to be involved in the glycolysis pathway (**Figure 4A**). Interestingly, the expression level of all DEGs associated with glycolysis was found to be higher in BM-BC compared to that in BM-LUAD (**Figure 4B**).

### Common upregulation of immune response modulation and tumor proliferation in brain metastasis of BC and LUAD

**Figure 5:**
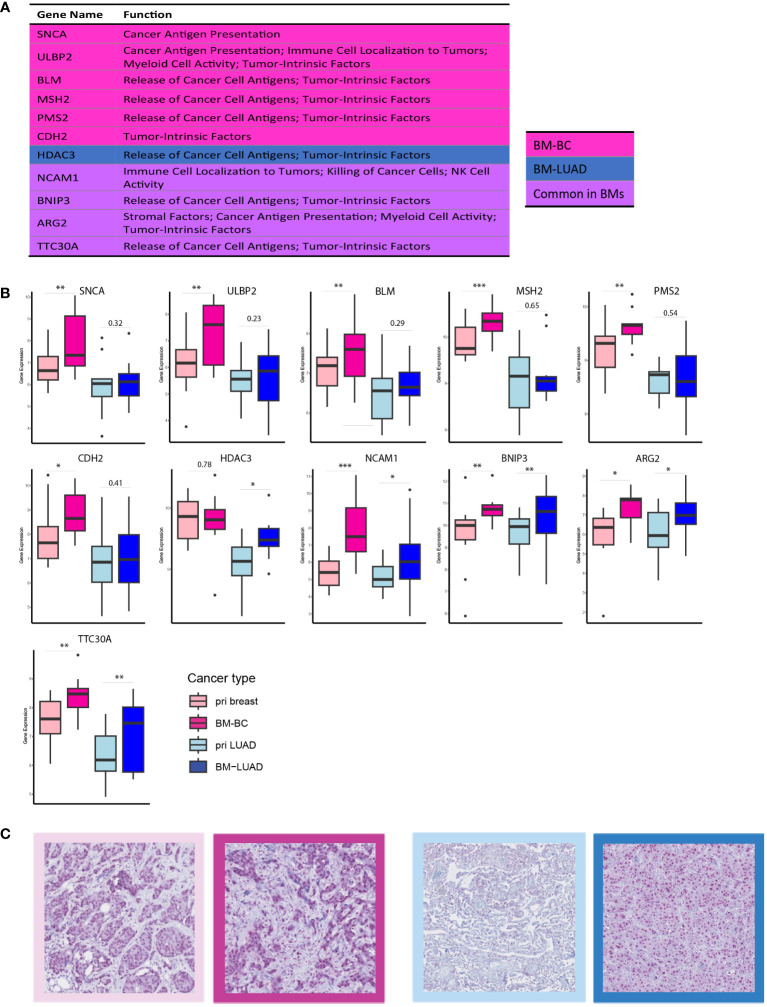
DEGs involved in immune response modulation in BM-BC and BM-LIUAD. **(A)** list of upregulated differentially expressed genes in BM-BC and BM-LUAD involved in immune response regulation. The *P-value* and adj. *P-value* of commonly upregulated differentially expressed genes are related to BM-BC. **(B)** Box plots of the differentially expressed genes involved in immune response regulation in brain metastasis (*adj. *p* ≤ 0.05, **adj. *p* ≤ 0.01, ***adj. *p* ≤ 0.001). **(C)** IHC staining for the expression of FDA-approved drug target HDAC3 in BC and LUAD paired samples. The red color in the IHC images indicates the expression of HDAC3 on tissue of BC and LUAD. The border colors of each image are as follow, light pink: primary BC, dark pink: BM-BC, light blue: primary LUAD, dark blue: BM-LUAD.

**Figure 6:**
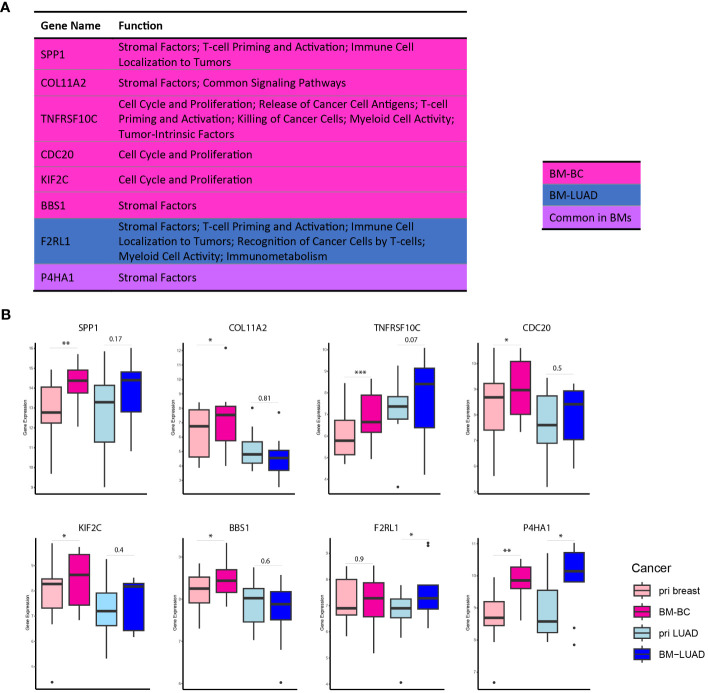
DEGs associated with tumor proliferation in BM-BC and BM-LIUAD. **(A)** list of upregulated differentially expressed genes in BM-BC and BM-LUAD associated with tumor growth and proliferation (adj. *p* ≤ 0.05). The *P-value* and adj. *P-value* of commonly upregulated differentially expressed genes are related to BM-BC. **(B)** Box plots of the differentially expressed genes associated with tumor growth and proliferation in brain metastasis (*adj. *p* ≤ 0.05, **adj. *p* ≤ 0.01, ***adj. *p* ≤ 0.001).

Further exploration of the upregulated DEGs in the brain metastases revealed their involvement in modulating the immune response. Thirty DEGs that were upregulated in BM-BC (excluding genes associated with metabolic stress) and 13 DEGs that were upregulated in BM-LUAD (excluding genes associated with metabolic stress) were included for GSA. A total of 11 genes were found to have function related to regulating (stimulation or inhibition) immune system in brain metastasis (**Figures 5A, B**). Importantly, an FDA-approved druggable target, HDAC3, was found to be upregulated in BM-LUAD as compared to primary LUAD (**Figure 5B**). These results were validated by IHC (**Figure 5C**). In addition, 8 genes were associated with tumor proliferation (**Figure 6A**). Remarkably, different types of tumors used distinct genes to regulate their proliferation (**Figures 6A, B**).

### Various potential druggable targets were expressed in the brain metastasis of different cancer types

**Figure 7:**
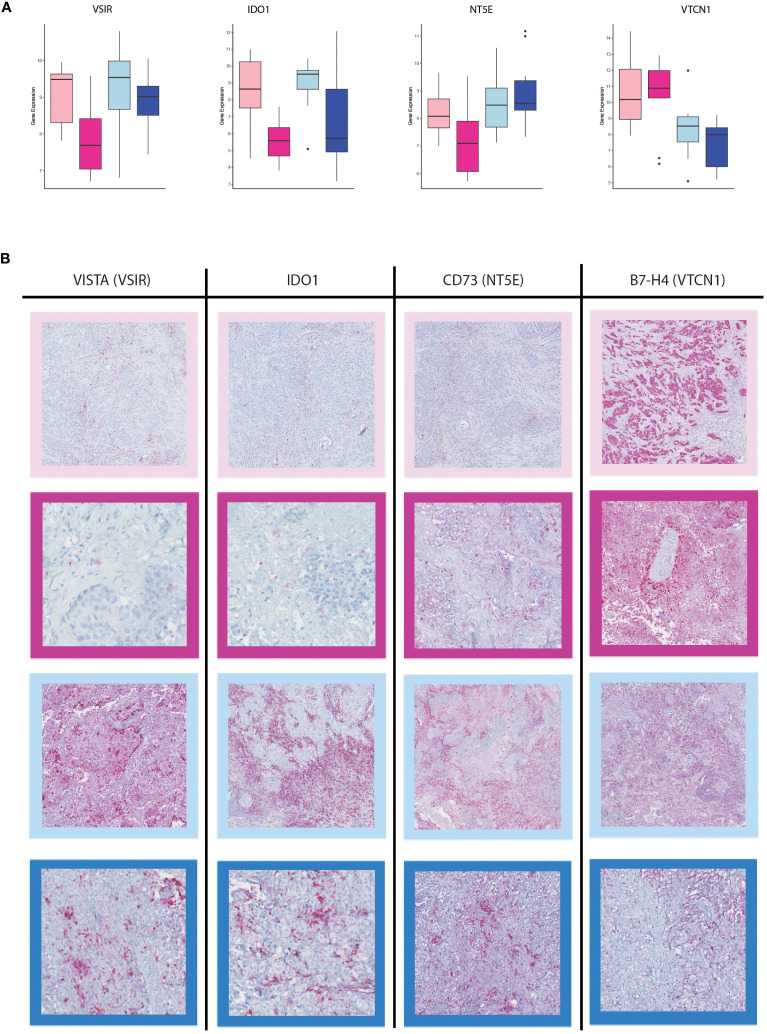
Immunohistochemistry staining for immune checkpoint inhibitors in the BM-BC and BM-LUAD samples. **(A)** Box plots of the differentially expressed immune checkpoint molecules in BM-BC and BM-LUAD (adj. *p* ≤ 0.05). **(B)** IHC staining for the expression of therapeutic targets VISTA, IDO1, NT5E (CD73), and VTCN1 (B7-H4) in BM-BC and BM-LUAD samples. The red color in each IHC image shows the expression of associated target on tissue of BC and LUAD. The border colors of each image are as follow, light pink: primary BC, dark pink: BM-BC, light blue: primary LUAD, dark blue: BM-LUAD.

A total of 12 immune checkpoint molecules were identified to be upregulated in primary tumors compared to their matched-paired brain metastases. However, only four immune checkpoint molecules exhibited significant differences of expression between the brain metastases from BC and LUAD (**Figure 7A**). The VTCN1 (B7-H4) was upregulated in BM-BC, whereas NT5E (CD73), VSIR and IDO1 were upregulated in BM-LUAD (**Figure 7A**). The expressional differences of these four immune checkpoints were validated at the protein level. Immunohistochemistry was performed in 2/11 breast cancer and 5/11 LUAD paired samples. Due to the amount of samples that was used for RNA extraction, the tumor compartment was lost in 9 breast cancer and 6 LUAD paired samples. Consistent with the gene expression results, higher expression of the immune checkpoint protein VTCN1 was observed in BM-BC compared to primary tumors and BM-LUAD (**Figure 7B**). Additionally, higher expression of the immune checkpoint proteins NT5E (CD73), VISTA (VSIR) and IDO1 was observed in primary tumors, with a predominant higher expression in BM-LUAD compared to BM-BC (**Figure 7B**).

## Discussion

Studying brain metastasis is important for the development of effective treatment options and ultimately improving patient outcomes (21). In the present study, we compared the primary tumors of breast and LUAD to their matched-paired brain metastases. We identified that cancer-related genes were upregulated in the primary tumors compared to their matched-paired brain metastases. In the brain metastases we found common upregulated genes suggestive of the presence of shared molecular mechanisms. In addition, we identified distinct differences of gene expression in BM-BC and BM-LUAD, signifying the utilization of unique mechanisms by different tumor types in brain metastasis. This finding highlights the heterogeneity that exists among brain metastases, revealing a complex interplay between common and unique molecular pathways in this context. Moreover, we found that the upregulated differentially expressed genes in BM-BC and BM-LUAD were associated with the metabolic stress pathway in brain metastasis, with a particular involvement of the glycolysis pathway. Interestingly, the majority of these genes exhibited higher upregulation in BM-BC compared to BM-LUAD, indicating higher glycolysis and glucose uptake in BM-BC. Limited studies have also provided evidence supporting the role of metabolic pathway in cancer metastasis. Dupuy et al. (22) showed that breast cancer cells with high metastatic potential undergo metabolic alterations leading to increased glycolysis and decreased mitochondrial metabolism. Parida et al. (23) showed that variations in metabolic pathways among brain-tropic cells influence their ability to adapt to the brain microenvironment and support tumor growth, highlighting the importance of understanding metabolic factors in brain metastasis. Tchou et al. (24) demonstrated that triple negative breast cancers (TNBCs) with high glycolytic rates exhibit an elevated proliferation index, indicating the association between glycolysis and tumor cell proliferation in this breast cancer subtype. These findings demonstrate the potential significance of metabolic pathway in promoting the survival and proliferation of metastatic cells within the brain microenvironment. However, it is imperative to validate these observations through functional studies, including experiments on glycolysis-related genes’ role in tumor metastasis, such as transwell or wound healing assays, in future research. Given our discovery of elevated glycolysis in BM-BC and the established literature on the impact of metabolic pathways on cancer progression, we hypnotize that the increased glycolysis in BM-BC may be influenced by dietary factors, particularly carbohydrate intake in daily food consumption. A comprehensive investigation into dietary habits and sugar intake patterns could offer valuable insights into the regulation of glycolysis in the context of BM-BC.

Furthermore, we found that the upregulated genes in BM-BC and BM-LUAD were involved in immune response regulation, tumor growth, and proliferation. Limited studies have utilized matched-paired samples to investigate immune related signatures. A study by Iwamoto et al. (16) involved gene expression analyses on 16 matched-paired samples between primary breast cancers and brain metastases. The results aimed to confirm previously reported genes associated with brain metastases and epithelial-mesenchymal transition (EMT) and identify novel therapeutic targets among FDA-approved agents or those investigated in clinical trials for distinct cancers. Their results revealed that immune-related signatures exhibited significantly lower gene expression in brain metastases than in primary breast cancers. Similar to our approach, Tsakonas et al. (2) employed immune-gene expression profiling of 13 primary lung samples and their matched-paired BM. However, detailed information on the specific subtype of the matched-paired samples is not available. Their results revealed significant downregulated of 12 immune-related genes in BM compared to primary tumors. Importantly, we found an FDA-approved druggable target (HDAC3, **Figures 5B, C**) that was differentially expressed in LUAD and was upregulated in BM-LUAD compared to primary LUAD. This finding suggests the potential for targeted therapy in BM-LUAD. In our previous study we discovered higher infiltration of immune cells in BM-LUAD as compared to BM-BC (19). Interestingly, despite the higher immune response regulation in BM-BC, we did not find significant immune infiltration between the primary tumors and their matched-paired brain metastases. This could be attributed to factors such as the poor blood-brain barrier (BBB) permeability, differences in genetic characteristics, or insufficient stimulation to facilitate additional infiltration. However, we discovered four immune checkpoint molecules that exhibited differential expression between BM-BC and BM-LUAD, suggesting their potential for developing lineage-specific therapeutic strategies for brain metastasis. Importantly, in line with our previous study, we found higher expression of VISTA and IDO1 in primary LUAD and BM-LUAD as compared to breast cancer, with a predominant higher expression in primary tumors. VISTA is an immune regulatory receptor expressed mainly by myeloid cells and is associated with poor overall survival in various cancers (25, 26). We previously found that VISTA is expressed on tumor cells, T lymphocytes, and to a lesser extent on microglia (19). The results highlight potential for targeted therapy strategies directed against VISTA in the treatment of brain metastasis originating from LUAD.

IDO1 is an immune response regulator involved in the suppression of the anti-tumor immune response and immune evasion. IDO1 is highly expressed in various malignant tumors including lung cancer, and its overexpression is linked to unfavorable clinical outcomes (27–30). Although we found higher expression of IDO1 in BM-LUAD, its level was relatively low as compared to VISTA. Nevertheless, the predominant expression of IDO1 in BM-LUAD should be taken into account regarding the development of combination therapies targeting IDO1. Similarly, the higher expression of IDO1 in 13 primary lung cancer and their matched-paired samples we discovered previously (2), confirming the results of our study. We also found higher expression of NT5E (CD73) in BM-LUAD than in BM-BC. CD73 is a transmembrane glycoprotein and exerts diverse functions in the tumor microenvironment (31). CD73 catalyzes the hydrolysis of adenosine, which contributes to cancer progression, neovascularization, tumor cell proliferation, and immune evasion (32). Moreover, CD73 has been found to be involved in invasion and metastasis across different cancers, such as ovarian and cervical carcinomas (32). Dual blockade of CD73-TGFβ has led to promote a complex inflammatory tumor microenvironment, characterized by decreased levels of myeloid-derived suppressor cells (MDSCs) and M2-macrophages, as well as increased levels of activated dendritic cells, cytotoxic T cells, and B cells (33). The prominent expression of CD73 in BM-LUAD, not in BM-BC, prompts this molecule as another target for the treatment of BM-LUAD. VTCN1, also known as B7-H4, is an immune regulatory molecule belonging to the B7 family (34). VTCN1 is mainly expressed by antigen-presenting cells (APCs) and induces immune suppression by inhibiting the expansion of neutrophil progenitors and T cells (35–37). Overexpression of VTCN1 in tumors correlates with their clinicopathological features, and promotes tumor proliferation, metastasis and immune evasion (38–43). We found higher expression of VTCN1 in BM-BCthan in their primary tumors, and also in BM-LUAD. The significant expression of VTCN1 in BM-BC should be considered as another potential target for inclusion in the development of targeted immunotherapy, especially in targeting BM-BC. In line with the previous article (44), we hypothesize that the immune system may play a role in the development of brain metastasis in breast and lung cancer. This particular immune system involvement requires additional validation, particularly in the case of lung adenocarcinoma.

There are some limitations in our study. The cohort size we analyzed was relatively small, however, it is important to highlight the exceptional uniqueness of the samples we had the opportunity to analyze. Utilizing matched-paired primary tumor and brain metastasis samples is an important and unique approach in cancer research, allowing for direct comparisons of molecular changes between the primary tumor and its corresponding brain metastasis. This not only unravels the specific adaptations that enable tumor cells to invade the brain but also sheds light on the mechanisms governing the selection and survival of metastatic cells in this unique microenvironment. Moreover, a control group consisting of tumors that did not metastasize to the brain was lacking for direct comparisons, however, our study provides valuable insights into various intriguing aspects of brain metastasis.

In conclusion, the comparison of molecular mechanism in primary breast and LUAD with their matched-paired brain metastases, provide valuable insights into the molecular landscape of brain metastasis. The findings of this study highlight the differential gene expression patterns, the significance of metabolic pathways specially the involvement of glycolysis pathway, and the impact of immune response modulation in brain metastasis. In addition, the identification of the FDA-approved druggable target (HDAC3) and the four immune checkpoint molecules in BM suggest that targeted therapies should be tailored for specific tumor lineages in order to effectively manage brain metastasis.

## Data availability statement

The data presented in the study are deposited in the https://www.ncbi.nlm.nih.gov/geo/query/acc.cgi?acc=GSE248830 repository, accession number GSE248830.

## Ethics statement

This study was approved by the Medical Ethics Committee of the Erasmus Medical Center, Rotterdam, the Netherlands and was carried out in adherence to the Code of Conduct of the Federation of Medical Scientific Societies in the Netherlands (MEC 02·953 & MEC-2020–0732). The studies were conducted in accordance with the local legislation and institutional requirements. The participants provided their written informed consent to participate in this study.

## Author contributions

SN: Data curation, Formal analysis, Investigation, Methodology, Resources, Software, Validation, Visualization, Writing – original draft. WK: Formal analysis, Writing – review & editing. JK: Conceptualization, Investigation, Project administration, Resources, Supervision, Writing – review & editing. DM: Conceptualization, Investigation, Project administration, Resources, Supervision, Writing – review & editing.
