# Supplementary material for: Unlocking molecular mechanisms and identifying druggable targets in matched-paired brain metastasis of breast and lung cancers
Source: Front Immunol. 2023 Dec 12;14:1305644. doi: 10.3389/fimmu.2023.1305644 (PMC10750385; doi:10.3389/fimmu.2023.1305644)
Supplement: Supplementary file 1 [file Table_1.docx]

**Table S1.** The list of housekeeping genes used for the normalization of genes.

| **N** | **Gene symbol** | **Gene name** | **SD** |
| --- | --- | --- | --- |
| 1 | UBB | Ubiquitin B | 0.659 |
| 2 | PUM1 | Pumilio RNA Binding Family Member 1 | 0.297 |
| 3 | TLK2 | Tousled Like Kinase 2 | 0.522 |
| 4 | DNAJC14 | DnaJ Heat Shock Protein Family (Hsp40) Member C14 | 0.696 |
| 5 | SF3A1 | Splicing Factor 3a Subunit 1 | 0.377 |
| 6 | POLR2A | RNA Polymerase II Subunit A | 0.563 |
| 7 | SDHA | Succinate Dehydrogenase Complex Flavoprotein Subunit A | 0.416 |
| 8 | STK11IP | Serine/Threonine Kinase 11 Interacting Protein | 0.607 |
| 9 | NRDE2 | NRDE-2, Necessary for RNA Interference, Domain Containing | 0.664 |
| 10 | TBP | TATA-Box Binding Protein | 0.438 |
| 11 | TBC1D10B | TBC1 Domain Family Member 10B | 0.368 |
| 12 | PSMC4 | Proteasome 26S Subunit, ATPase 4 | 0.574 |
| 13 | ABCF1 | ATP Binding Cassette Subfamily F Member 1 | 0.56 |
| 14 | TMUB2 | Transmembrane And Ubiquitin Like Domain Containing 2 | 0.471 |
| 15 | MRPL19 | Mitochondrial Ribosomal Protein L19 | 0.409 |
| 16 | ERCC3 | ERCC Excision Repair 3, TFIIH Core Complex Helicase Subunit | 0.377 |

*Note.* Order of HKGs is selected by geNorm algorithm of nSolver; SD: standard deviation after normalization.

**Table S2.** Details of antibodies used in immunohistochemistry staining.

| **N** | **Antibody** | **Type** | **Concentration** | **Manufactured by** | **Clone** |
| --- | --- | --- | --- | --- | --- |
| 1 | VISTA | Anti-Rabbit | 1/800 | Novusbio | Monoclonal |
| 2 | IDO1 | Anti-Rabbit | 1/500 | Abcam | Monoclonal |
| 3 | NT5E | Anti-Rabbit | 1/800 | Abcam | Monoclonal |
| 4 | VTCN1 | Anti-Rabbit | 1/100 | Abcam | Monoclonal |
| 5 | HDAC3 | Anti-Rabbit | 1/100 | Abcam | Monoclonal |

*Note.* VISTA: V-domain Ig suppressor of T cell activation; IDO1: Indoleamine 2; NT5E: ecto-5′-nucleotidase; VTCN1: V-Set Domain Containing T Cell Activation Inhibitor 1; HDAC3: Histone deacetylase 3.
